# Neurological features of Hansen disease: a retrospective, multicenter cohort study

**DOI:** 10.1038/s41598-024-60457-0

**Published:** 2024-05-06

**Authors:** Xiaohua Chen, Li Di, Min Qian, Dongchao Shen, Xinhong Feng, Xiqing Zhang

**Affiliations:** 1 Leprosy Department, Beijing Tropical Medicine Research Institute, Beijing Friendship Hospital, Capital Medical University, Beijing, China; 2 Beijing Key Laboratory for Research On Prevention and Treatment of Tropical Diseases, Capital Medical University, Beijing, China; 3 Department of Neurology, Beijing Xuanwu Hospital, Capital Medical University, Beijing, China; 4 Department of Neurology, Peking Union Medical College Hospital (PUMCH), Chinese Academy of Medical Sciences and Peking Union Medical College (CAMS & PUMC), Beijing, China; 5 Department of Neurology, School of Clinical Medicine, Beijing Tsinghua Changgung Hospital, Tsinghua University, Beijing, China; 6 Department of Neurology, Beijing Junyi Traditional Chinese Medicine Hospital, Beijing, China

**Keywords:** Diseases, Medical research, Neurology, Signs and symptoms

## Abstract

To elucidate the neurological features of Hansen disease. The medical records of patients with confirmed Hansen disease transferred from the neurology department were reviewed, and all medical and neurological manifestations of Hansen disease were assessed. Eleven patients with confirmed Hansen disease, 10 with newly detected Hansen disease and 1 with relapsed Hansen disease, who visited neurology departments were enrolled. The newly detected patients with Hansen disease were classified as having lepromatous leprosy (LL, n = 1), borderline lepromatous leprosy (BL, n = 2), borderline leprosy (BB, n = 2), borderline tuberculoid leprosy (BT, n = 1), tuberculoid leprosy (TT, n = 2), or pure neural leprosy (PNL, n = 2). All of the patients with confirmed Hansen were diagnosed with peripheral neuropathy (100.00%, 11/11). The symptoms and signs presented were mainly limb numbness (100.00%, 11/11), sensory and motor dysfunction (100.00%, 11/11), decreased muscle strength (90.90%, 10/11), and skin lesions (81.81%, 9/11). Nerve morphological features in nerve ultrasonography (US) included peripheral nerve asymmetry and segmental thickening (100.00%, 9/9). For neuro-electrophysiology feature, the frequency of no response of sensory nerves was significantly higher than those of motor nerves [(51.21% 42/82) vs (24.70%, 21/85)(P = 0.0183*)] by electrodiagnostic (EDX) studies. Nerve histological features in nerve biopsy analysis included demyelination (100.00%, 5/5) and axonal damage (60.00%, 3/5). In addition to confirmed diagnoses by acid-fast bacteria (AFB) staining (54.54%, 6/11) and skin pathology analysis (100.00%, 8/8), serology and molecular technology were positive in 36.36% (4/11) and 100.00% (11/11) of confirmed patients of Hansen disease, respectively. It is not uncommon for patients of Hansen disease to visit neurology departments due to peripheral neuropathy. The main pathological features of affected nerves are demyelination and axonal damage. The combination of nerve US, EDX studies, nerve biopsy, and serological and molecular tests can improve the diagnosis of Hansen disease.

## Introduction

Leprosy, also known as Hansen's disease, is caused by *Mycobacterium leprae* infection and affects mainly the skin and peripheral nervous system. Neuropathy is an integral symptom of Hansen disease^1^. Hansen disease is associated with neuropathy, which results in nerve function impairment and causes disabilities^2^. Neuropathy and its related disabilities are the major medical consequences of Hansen disease and remain a global medical concern^3^.

Hansen disease is one of the most common treatable peripheral neuropathies in the world^4^. *Mycobacterium leprae* (and *M. lepromatosis*) is the only pathogenic bacteria able to infect peripheral nerves^5^. Antimicrobial therapy is effective^3^, and early treatment is associated with good outcomes^4^; however, neuropathy remains a problem, especially if diagnosis and treatment are delayed^3^. Neural impairment results in a set of sensory, motor and autonomic disturbances^5^. Despite major advances in understanding the mechanisms of *M. leprae* entry into peripheral nerves, most aspects of the pathogenesis of Hansen disease neuropathy are poorly understood^3^.

According to the distinct clinical manifestations and immunological spectra of the disease, Ridley and Jopling classified Hansen disease into five polar forms: tuberculoid (TT), borderline tuberculoid (BT), borderline (BB), borderline lepromatous (BL), and lepromatous (LL)^6^. For treatment purposes, the World Health Organization (WHO) currently classifies Hansen disease cases into two groups according to the number of skin lesions: multibacillary (MB) and paucibacillary (PB)^7^. Pure neural leprosy (PNL) is a rare clinical form of Hansen disease in which patients do not present with classic skin lesions but have a high burden of disability associated with the disease^8^. Dermatologic clinical manifestations of Hansen disease are highly variable and are described as “great imitators”^9^, while the clinical neurological manifestations of Hansen disease are still unclear.

In this study, we retrospectively described the neurological clinical data of patients with confirmed Hansen disease transferred from the neurology department. We hope that this work will provide further insight into the clinical characteristics of Hansen disease in neurology.

## Results

### Clinical characteristics of patients with Hansen disease who visited the neurology department

A total of 11 patients (6 males, 5 females; mean age 43.55 ± 13.13 years) with confirmed Hansen disease who initially visited and/or were referred to the neurology department during the study period were evaluated (Table 1). The patients with Hansen disease were of Han nationality and were from 9 provinces in different endemic regions [Northeast: Heilongjiang and Jilin; North: Hebei; East: Anhui; Southwest: Hubei, Hunan, Sichuan, and Yunnan; and Northwest: Shaanxi and Xinjiang] in China. The main complaint patients mainly presented with was limb numbness (63.63%, 7/11), inability to walk (18.18%, 2/11), and inability to lift their finger (18.18%, 2/11). Through physical examination, obvious skin lesions were found in 81.81% (9/11) of the patients, and a skin numbness area was found in 18.18% (2/11) of the patients. Through consultation, only one patient (9.09%, 1/11) was diagnosed with newly detected Hansen disease, while 90.90% (10/11) of the patients denied a history of contact with an individual with Hansen disease.

**Table 1:** The demographic and clinical characteristics of the confirmed patients with Hansen disease enrolled in this study.

Patient	Sex	Age (years)	Nationality	Place of ancestral/ residence	Education	Career	Main complaint
1	Male	45	Han	Shaanxi, China	Middle school	Worker	Loss of sensation in limbs for 7 years
2	Male	29	Han	Anhui, China	Middle school	Worker	Foot numbness for 10 + years, hand numbness for 3 + years
3	Male	66	Han	Heilongjiang, China	College	Retired	Limping for 25 years
4	Female	56	Han	Hunan,China	Primary School	Farmer	Left lower limb numbness for 7 years, limb numbness for 6 months
5	Female	47	Han	Sichuan,China	Middle school	Officer	Pain and coldness in both lower limbs for 2 years, limb numbness and weakness for 18 months
6	Male	46	Han	Hubei,China	College	Businessman	Numbness of the right hand that gradually invaded the limbs for 18 years,
7	Female	29	Han	Yunnan/Hebei, China	Middle school	Housewife	Right 4th and 5th finger numbness with limited activity for more than 1 year
8	Male	28	Han	Hubei,China	College	Officer	Inability to raise fingers of the right hand, which gradually progressed to the entire palm, for 11 years
9	Female	41	Han	Jilin,China	Middle school	Officer	Left foot numbness and pain for 1 month, left hand pain 24 days
10	Female	27	Han	Shaanxi, China	College	Officer	Right foot drop for half a year
11	Male	54	Han	Sichuan/Xinjiang, China	Middle school	Worker	Weakness in both lower limbs with an inability to walk for 6 years, clincally cured leprosy

### Neurologic evaluation

Sensory and motor nerve dysfunction was evaluated by neurologists at the general hospital (Table 2). Shallow sensation deficits occurred in all patients (100.00%, 11/11). Muscle weakness caused by peripheral neuropathy occurred in 81.82% (9/11) of the patients. Froment's sign was positive in 45.45% (5/11) of the patients. Steppage gait was observed in 36.36% (4/11) patients.

**Table 2:** Results of neurologic examination for confirmed patients with Hansen disease who visited the neurology department.

Patients			1		2		3		4		5		6		7		8		9		10		11	
			L	R	L	R	L	R	L	R	L	R	L	R	L	R	L	R	L	R	L	R	L	R
Muscle strength	Upper limbs	Shoulder abduction	V	V	V	V	V	V	V	V	V	V	V	V	V	V	V	V	V	V	V	V	V	V
		Elbow flexion	V	V	V	V	V	V	V	V	V	V	V	V	V	V	V	V	V	V	V	V	V	V
		Elbow extension	V	V	V	V	V	V	V	V	V	V	V	V	V	V	V	**IV**	V	V	V	V	V	V
		Wrist flexion	V	V	V	V	V	V	V	V	**IV–**	**IV + **	V	V	V	V	V	**IV**	V	V	V	V	V	V
		Wrist extension	V	V	V	V	V	V	V	V	V	V	V	V	V	V	V	**IV**	V	V	V	V	V	V
		Finger flexion	**IV**	**IV**	V	V	V	V	V	V	V	V	V	**IV**	V	**III**	V	V	V	V	V	V	V	V
		Finger extension	**IV**	**IV**	V	V	V	V	V	V	**II**	**II**	V	V	V	**III**	V	**IV**	V	V	V	V	V	V
		Fingers apart	**IV**	**IV**	V	V	V	V	V	V	**II**	**II**	**III**	**I**	V	**IV**	V	**III**	V	V	V	V	V	V
		Fingers together	**IV**	**IV**	V	V	V	V	V	V	**II**	**II**	**III**	**I**	**IV**	**IV**	V	**III**	V	V	V	V	V	V
		Froment's sign	**(+)**	**(+)**	(−)	(−)	(−)	(−)	(−)	(−)	**(+)**	**(+)**	**(+)**	**(+)**	**(+)**	**(+)**	(−)	**(+)**	(−)	(−)	(−)	(−)	(−)	(−)
	Lower limbs	Hip flexion	V	V	V	V	V	V	V	V	V	V	V	V	V	V	V	V	V	V	V	V	V	V
		Hip extension	V	V	V	V	V	V	V	V	V	V	V	V	V	V	V	V	V	V	V	V	V	V
		Bend flexion	V	V	V	V	V	V	**IV**	V	V	V	V	V	V	V	V	V	V	V	V	V	V	V
		Bend extension	V	V	V	V	V	V	**IV**	V	V	V	V	V	V	V	V	V	V	V	V	V	V	V
		Dorsalis pedis flexion	V	V	V	V	V	V	**IV**	V	V	V	V	**I**	**IV**	V	V	V	V	V	V	**III**	**III-IV**	**V–**
		Plantar flexion	V	V	V	V	**III**	**III**	**IV**	V	**II**	**IV–**	V	**I**	**IV**	V	V	V	V	V	V	**0**	**III-IV**	**V–**
		Toes flexion	V	V	V	V	**III**	**III**	**IV**	V	V	V	V	V	**IV**	V	V	V	V	V	V	**III**	**III-IV**	**V–**
		Toes extension	V	V	V	V	**III**	**III**	**IV**	V	V	V	V	V	**IV**	V	V	V	V	V	V	**0**	**III-IV**	**V–**
		Steppage **gait**	(−)		(−)		**(+)**		**(+)**		(−)		**(+)**		(−)		(−)		(−)		(−)		**(+)**	
Diminished skin shallow sensation		Upper limbs	**(+)**	**(+)**	**(+)**	**(+)**	(−)	(−)	**(+)**	**(+)**	**(+)**	**(+)**	**(+)**	**(+)**	(−)	**(+)**	(−)	**(+)**	(−)	**(+)**	(−)	(−)	**(+)**	**(+)**
		Lower limbs	**(+)**	**(+)**	**(+)**	**(+)**	**(+)**	**(+)**	**(+)**	**(+)**	**(+)**	**(+)**	**(+)**	**(+)**	**(+)**	**(+)**	(−)	(−)	**(+)**	(−)	(−)	**(+)**	**(+)**	**(+)**
		Skin lesions	(−)	(−)	**(+)**	**(+)**	**(+)**	**(+)**	**(+)**	**(+)**	**(+)**	**(+)**	**(+)**	**(+)**	(−)	(−)	(−)	(−)	(−)	(−)	(−)	(−)	**(+)**	**(+)**

Significant values are in bold.

*L* left, *R* right, (+) positive, (−) negative.

### Neurosonography findings

The peripheral nerves of nine patients were evaluated via ultrasonography (Table 3). Nerve cross-sectional area (CSA) was bilaterally measured via ultrasonography. There was a significant increase in the CSA of the affected nerves. The peripheral nerve form features revealed by ultrasonography also included asymmetrical and segmental thickening in all of the patients (100.00%, 9/9); swelling in 4 patients (44.44%, 4/9); decreased echo intensity in nerve bundles in 4 patients (44.44%, 4/9); and increased echo intensity in the epineurium in 2 patients (22.22%, 2/9). Good continuity of the nerve was observed in 66.67% (6/9) of the patients, and blood flow, as measured by color Doppler blood flow imaging (CDFI), was increased in 4 patients (44.44%, 4/9).

**Table 3:** Results of neurosonography for confirmed patients with Hansen disease who visited the neurology department.

Nerve ultrasonography			Patient 1	Patient 3	Patient 5	Patient 6	Patient 7	Patient 8	Patient 9	Patient 10	Patient 11
CSA (mm2)	Brachial plexus	Left	ND	ND	5–8 (Ref)	5–7 (Ref)	ND	11–16 (Ref)	ND	ND	4.3–10.8 (Ref)
		Right	ND	ND	5–8 (−)	6–9 (−)	ND	13–16 (−)	ND	ND	6.3–10.9 (–)
	Ulnar	Left	ND	ND	6–9 (Wrist, Ref) 13–18 (Elbow↑)	5–9 (Ref)	ND	3–5 (Ref)	4–11 (Ref)	ND	4.9–13 (Wrist, Ref) 7.9–21.4(Elbow↑)
		Right	ND	ND	4–7 (Wrist, Ref) 6–14 (Elbow↑)	7–31 (↑)	φ4.0 (↑)	7–9 (↑)	11–23 (↑)	ND	5.5–7.9 (Wrist, Ref) 11.2- 14.6 (Elbow↑)
	Median	Left	ND	ND	6–8 (Elbow, Ref) 5–18 (Wrist ↑)	6–9 (Ref)	10–16 (Ref)(↑)	6–11 (Ref)	ND	ND	7.9–10.1 (Elbow, Ref) 5.7–13.2 (Wrist↑)
		Right	ND	ND	5–5 (Elbow, Ref) 5–19 (Wrist↑)	8–28 (↑)	8–19 (↑)	7–48 (↑)	ND	ND	9.4–11.9 (Elbow, Ref) 6.2–11.7 (Wrist↑)
	Radial	Left	ND	ND	ND	ND	ND	10 (Ref)	ND	ND	ND
		Right	ND	ND	ND	ND	ND	17 (↑)	ND	ND	ND
	Sciatic nerve	Left	ND	ND	ND	ND	74–136 (Ref)	ND	ND	88–157 (Ref)	ND
		Right	ND	ND	ND	ND	80–139(-)	ND	ND	84–167(−)	ND
	Common peroneal	Left	68 (↑)	21–23 (↑)	ND	ND	19–35 (↑)	18–21 (Ref)	9–13 (Ref)	8 (Ref)	ND
		Right	Ref (↑)	14–18 (Ref)	ND	ND	16–26 (Ref)	15–23 (−)	9–12 (−)	17 (↑)	ND
	Tibial	Left	Ref (↑)	ND	ND	ND	ND	ND	ND	12–13 (Ref)	ND
		Right	68 (↑)	ND	ND	ND	ND	ND	ND	13–17 (↑)	ND
Asymmetrical and segmental thickening			**(+)**	**(+)**	**(+)**	**(+)**	**(+)**	**(+)**	**(+)**	**(+)**	**(+)**
Nerve swelling			**(+)**	ND	ND	ND	**(+)**	ND	**(+)**	**(+)**	ND
Echo intensity	Nerve bundles		↓	Homogeneous	ND	ND	↓	Homogeneous	↓	↓	ND
	Epineurium		ND		ND	ND	ND		↑	↑	ND
Continuity			Good	Good	ND	ND	Good	Good	Good	Good	ND
CDFI			↑	ND	ND	↑	No	↑	No	No	↑

*CSA* cross-sectional area, *CDFI* color Doppler blood flow imaging, *ND* not described.

*Ref* reference; (+): positive; (−): negative; up arrow (↑): increased; down arrow (↓): decreased.

### Electrodiagnostic findings

The nerve conduction velocity (NCV), F-wave, H reflex, skin sympathetic response (SSR) and needle electromyography (EMG) were performed for 10 patients (Table 4). The ulnar (20/20 & 20/20), median (20/20 & 20/20), radial (5/20 & 5/20), common peroneal (20/20 & 19/20), and tibial (20/20 & 18/20) peripheral nerves were tested for motor and sensory NCV, respectively.

**Table 4:** The electrodiagnostic study results for the confirmed patients with Hansen disease enrolled in this study.

Patients	NCV		Motor			Sensory			F-wave	H-reflex	SSR	EMG
			Latency	Amplitude	Conduction velocity	Latency	Amplitude	Conduction velocity				
Patient 1	Ulnar	Left	No Response			No Response			No response of bilateral median nerve and bliateral posterior tibia nervel	Normal	No Response	Neurogenic damage
	(Wrist)	Right	No Response			No Response						
	Median	Left	No Response			No Response						
	(Wrist)	Right	No Response			No Response						
	Radial	Left	ND	ND	ND	ND	ND	ND				
	(Elbow)	Right	ND	ND	ND	ND	ND	ND				
	Common peroneal	Left	Normal	0.659↓	Normal	No Response						
	(Fibular)	Right	Normal	1.568↓	Normal	No Response						
	Tibial	Left	No Response			No Response						
	(Ankle)	Right	No Response			No Response						
Patient 3	Ulnar	Left	Normal	Normal	Normal	Normal	3.6↓ 81%	39↓ 28%	Prolonged latency of right tibial nerve. Axonal reflex	Normal	ND	Neurogenic damage
	(Wrist)	Right	Normal	Normal	Normal	Normal	2.0↓ 89%	-				
	Median	Left	Normal	5.9↓61%	Normal	Normal	9.4↓ 69%	37↓26%				
	(Wrist)	Right	Normal	4.7↓69%	Normal	Normal	5.3↓ 82%	33↓34%				
	Radial	Left	ND	ND	ND	ND	ND	ND				
	(Elbow)	Right	ND	ND	ND	ND	ND	ND				
	Common peroneal	Left	Normal	0.2↓ 96%	35↓ 39%	Normal	Normal	38↓33%				
	(Ankle)	Right	Normal	0.4↓ 93%	32↓ 44%	Normal	0.7↓ 84%	36↓ 36%				
	Tibial	Left	Normal	Normal	Normal	Normal	Normal	Normal				
	(Ankle)	Right	Normal	Normal	Normal	Normal	Normal	Normal				
Patient 4	Ulnar	Left	Normal	Normal	Normal	Normal	Normal	Normal	Normal	Normal	ND	Neurogenic damage
	(Wrist)	Right	Normal	Normal	Normal	Normal	Normal	Normal				
	Median	Left	Normal	Normal	Normal	No Response						
	(Wrist)	Right	Normal	Normal	Normal	Normal	Normal	Normal				
	Radial	Left	ND	ND	ND	ND	ND	ND				
	(Elbow)	Right	ND	ND	ND	ND	ND	ND				
	Common peroneal	Left	No Response			No Response						
	(Ankle)	Right	5.1↑95%	Normal	41 LLN*	Normal	Normal	Normal				
	Tibial	Left	No Response			No Response						
Patient 5	(Ankle)	Right	Normal	Normal	Normal	No Response						
	Ulnar	Left	No Response			No Response			Normal	ND	ND	Neurogenic damage
	(Wrist)	Right	No Response			No Response						
	Median	Left	No Response			No Response						
	(Wrist)	Right	No Response			No Response						
	Radial	Left	ND	ND	ND	ND	ND	ND				
	(Elbow)	Right	ND	ND	ND	ND	ND	ND				
	Common peroneal	Left	No Response			ND	ND	ND				
	(Ankle)	Right	No Response			No Response						
	Tibial	Left	No Response			ND	ND	ND				
	(Ankle)	Right	No Response			No Response						
Patient 6	Ulnar	Left	Normal	Normal	Normal	Normal	Normal	Normal	Normal	ND	ND	Neurogenic damage
	(Wrist)	Right	No Response			No Response						
	Median	Left	Normal	Normal	Normal	Normal	Normal	Normal				
	(Wrist)	Right	Normal	0.07↓ 99%	Normal	No Response						
	Radial	Left	ND	ND	ND	ND	ND	ND				
	(Elbow)	Right	Normal	Normal	Normal	No Response						
	Common peroneal	Left	No Response			No Response						
	(Ankle)	Right	No Response			No Response						
	Tibial	Left	Normal	Normal	Normal	No Response						
	(Ankle)	Right	Normal	Normal	Normal	ND	ND	ND				
Patient 7	Ulnar	Left	Normal	Normal	Normal	Normal	Normal	Normal	Normal	Normal	No Response	Neurogenic damage
	(Wrist)	Right	Normal	1.815↓ 90%	Normal	No Response						
	Median	Left	Normal	Normal	Normal	Normal	Normal	Normal				
	(Wrist)	Right	Normal	Normal	Normal	No Response						
	Radial	Left	ND	ND	ND	ND	ND	ND				
	(Elbow)	Right	ND	ND	ND	ND	ND	ND				
	Common peroneal	Left	6.1↑133%	0.047↓ 99%	27↓ 57%	No Response						
	(Ankle)	Right	11.5↑ 339%	1.074↓ 80%	Normal	No Response						
	Tibial	Left	6.2↑59%	1.491↓ 92%	Normal	No Response						
	(Ankle)	Right	5.2↑ 33%	3.356↓ 82%	Normal	No Response						
Patient 8	Ulnar	Left	Normal	Normal	Normal	Normal	Normal	Normal	Normal	Normal	ND	Neurogenic damage
	(Wrist)	Right	Normal	Normal	Normal	Normal	Normal	Normal				
	Median	Left	Normal	Normal	Normal	Normal	14.8↓ 79%	Normal				
	(Wrist)	Right	Normal	2.6↓ 87%	36↓44%	No Response						
	Radial	Left	Normal	Normal	Normal	Normal	Normal	Normal				
	(Elbow)	Right	Normal	6.8↓ 51%	5.2↓90%	Normal	4.0↓ 92%	Normal				
	Common peroneal	Left	Normal	1.7↓ 47%	Normal	Normal	Normal	Normal				
	(Ankle)	Right	Normal	Normal	Normal	Normal	Normal	Normal				
	Tibial	Left	Normal	Normal	Normal	Normal	Normal	Normal				
	(Ankle)	Right	Normal	Normal	Normal	Normal	Normal	Normal				
Patient 9	Ulnar	Left	Normal	Normal	Normal	No Response			Normal	Normal	ND	Neurogenic damage
	(Wrist)	Right	Normal	2.7↓ 85%	55↓18%	Normal	Normal	Normal				
	Median	Left	Normal	Normal	Normal	Normal	Normal	Normal				
	(Wrist)	Right	Normal	Normal	Normal	Normal	19.5↓ 44%	Normal				
	Radial	Left	ND	ND	ND	ND	ND	ND				
	(Elbow)	Right	ND	ND	ND	ND	ND	ND				
	Common peroneal	Left	Normal	0.5↓94%	42 LLN	Normal	Normal	Normal				
	(Ankle)	Right	Normal	1.6↓ 80%	42 LLN	Normal	Normal	Normal				
	Tibial	Left	Normal	5.0↓ 64%	Normal	No Response						
	(Ankle)	Right	Normal	Normal	Normal	Normal	Normal	Normal				
Patient 10	Ulnar	Left	Normal	Normal	Normal	Normal	Normal	Normal	Normal	Normal	ND	Neurogenic damage
	(Wrist)	Right	Normal	Normal	46↓ 29%	Normal	8.9↓ 58%	4.2↓ 28%				
	Median	Left	Normal	Normal	Normal	Normal	Normal	Normal				
	(Wrist)	Right	Normal	Normal	50↓ 22%	Normal	7.7↓ 73%	44↓ 28%				
	Radial	Left	Normal	Normal	Normal	Normal	Normal	Normal				
	(Elbow)	Right	Normal	Normal	Normal	Normal	Normal	Normal				
	Common peroneal	Left	Normal	Normal	Normal	Normal	Normal	Normal				
	(Ankle)	Right	Normal	0.2↓ 96%	Normal	No Response						
	Tibial	Left	Normal	Normal	Normal	No Response						
	(Ankle)	Right	Normal	Normal	Normal	No Response						
Patient 11	Ulnar	Left	Normal	Normal	Normal	No Response			Prolonged latency and decreased F-wave frequency of left ulnar nerve. Axonal damage & demyelination	ND	ND	Neurogenic damage
	(Wrist)	Right	Normal	Normal	Normal	No Response						
	Median	Left	Normal	Normal	Normal	No Response						
	(Wrist)	Right	Normal	Normal	Normal	No Response						
	Radial	Left	ND	ND	ND	ND	ND	ND				
	(Elbow)	Right	ND	ND	ND	ND	ND	ND				
	Common peroneal	Left	No Response			No Response						
	(Ankle)	Right	Normal	1.7↓	Normal	No Response						
	Tibial	Left	No Response			Normal	Normal	Normal				
	(Ankle)	Right	Normal	Normal	Normal	Normal	Normal	Normal				

*NCV* nerve conduction velocity, *SSR* skin symptomatic response, *EMG* electromyography, *ND* not described, *LLN* lower limit of normal.

The main electrodiagnostic (EDX) findings were as follows: (1) both motor and sensory fibers of peripheral nerves were affected in 100.00% of the patients (10/10); (2) asymmetrical nerve damage was present in not only motor but also sensory fibers in 100.00% of the patients (10/10); (3) motor fibers were most frequently affected in the common peroneal (90.00%, 18/20), tibial (55%, 11/20), median (40.00%, 8/20), ulnar (35%, 7/20), and radial (20.00%, 1/5) nerves; (4) sensory fibers were most frequently affected in the common peroneal (63.15%, 12/19), tibial (61.11%, 11/18), ulnar (60.00%, 12/20), median (50.00%, 10/20), and radial (40.00%, 2/5) nerves; (5) sensory fibers (57.31%, 47/82) and motor fibers (52.94%, 45/85) were similarly affected (P = 0.6997); and (6) sensory [62.16% ((23/37)) vs 53.33% (24/45)](P = 0.7162) and motor [72.50% (29/40) vs 35.55% (16/45)] fibers of the lower limbs and upper limbs were similarly affected (P = 0.0669).

The NCV findings were as follows (Table 5): (1) “No response” was the main feature for both sensory and motor fibers and occurred in more sensory fibers (51.21%, 42/82) than motor fibers (24.70%, 21/85) (P = 0.0183*). (2) “Decreased amplitude” was the secondary feature [24.70% (21/85) vs. 12.19% (10/82)] (P = 0.1160), followed by “decreased conduction velocity” [9.41% (8/85) vs. 8.53% (7/82)] (P > 0.9999), and “prolonged distal latency” [5.88% (5/85) vs. 0.00% (0/82)] (P = 0.0602) for both motor and sensory fibers. Moreover, there were no significant differences between motor and sensory fibers.

**Table 5:** The nerve conduction velocity of the confirmed patients with Hansen disease enrolled in this study.

Nerves		Motor (n, %)		Sensory (n, %)		P value (motor vs sensory)
Affected/Tested nerves (n, %)	Upper limbs	16/45	35.55%	24/45	53.33%	0.3432
	Ulnar	7/20	35.00%	12/20	60.00%	0.4093
	Median	8/20	40.00%	10/20	50.00%	0.7804
	Radial	1/5	20.00%	2/5	40.00%	> 0.9999
	Lower limbs	29/40	72.50%	23/37	62.16%	0.7209
	Common peroneal	18/20	90.00%	12/19	63.15%	0.6285
	Tibial	11/20	55.00%	11/18	61.11%	> 0.9999
	Total	45/85	52.94%	47/82	57.31%	0.6997
	P value (upper vs lower)	0.0669		0.7162		
NCV	No response	21/85	24.70%	42/82	51.21%	0.0183*
	Prolonged latency	5/85	5.88%	0/82	0.00%	0.0602
	Decreased amplitude	21/85	24.70%	10/82	12.19%	0.1160
	Decreased conduction velocity	8/85	9.41%	7/82	8.53%	> 0.9999
	Low limit of normal (LLN)	3/85	3.52%	0/82	0.00%	0.2465

*NCV* Nerve conduction velocity.

*P < 0.05.

In addition, F-wave abnormalities were found in 3 patients; no response was observed in 1 patient, prolonged latency was observed in 2 patients, and a decreased frequency was observed in 1 patient.The H-reflex was normal in 7 patients. However, SSRs were not detected in 2 patients. EMG demonstrated neurogenic damage in 10 patients (Table 4).

### Nerve and skin biopsy findings

Nerve biopsy was performed for 6 patients (Table 6). In hematoxylin–eosin (HE) staining, the myelin sheath and axon were damaged or absent in 66.66% (4/6) of the patients, while Schwann cells were hyperplastic in 50.00% (3/6) of the patients but were absent in 16.67% (1/6) of the patients. Inflammation was absent in 50.00% (3/6) of patients and was present in 50.00% (3/6) of patients. Tissue cell infiltration was observed in 33.33% (2/6) patients. Fibrous tissue of the nerve perineurium presented hyperplasia in 16.67% (1/6) of patients. Edema and mucus denaturation presenting as hyperplasia were observed in 16.67% (1/6) of patients.

**Table 6:** The nerve biopsy results of confirmed patients with Hansen disease enrolled in this study.

Nerve Pathology	Patients		3	5	6	7	10	11
	Nerve biopsy		Sural nerve					
Hematoxylin–eosin staining (HE)	Myelin sheath		No definite absence	Destruction	Destruction	Destruction	Destruction	ND
	Axons		No definite absence	Destruction	Destruction	Destruction	Absent	ND
	Schwann cell		ND	Hyperplasia	Hyperplasia	Absent	Hyperplasia	ND
	Inflammatory cells		Absent	Infiltrates	Absent	Infiltrates	Absent	Infiltrates
	Tissue cells		ND	Infiltrates	Infiltrates	ND	ND	ND
	Fibrous tissue of nerve perineurium		ND	ND	ND	Hyperplasia	ND	ND
	Edema & mucus denaturation		ND	ND	ND	ND	ND	Present
Special staining	Luxol fast blue(LFB)		ND	ND	**(−)**	ND	ND	ND
	Masson staining		ND	ND	ND	**(+)**	ND	**(−)**
	Congo red staining		ND	ND	ND	ND	ND	**(−)**
	Toluidine blue staining		ND	ND	ND	ND	ND	**(−)**
	Acid-fast staining		ND	ND	ND	**(−)**	ND	**(−)**
	Weak acid-fast staining		ND	**(+)**	ND	ND	ND	**(−)**
Immunohistochemistry (ICH)	Myelin sheath	MBP	**(+)**	ND	ND	**(−)**	**(+)**	**(+)**
		LFB + HE	**(+)**	ND	ND	**(−)**	**(−)**	ND
	Axles	NF	**(+)**	ND	ND	**(−)**	**(+)**	ND
	Schwann cell	SOX-10	**(+)**	ND	ND	**(+)**	**(+)**	ND
	Nerve	S-100	ND	**(+)**	ND	ND	ND	**(+)**
	Macrophage	CD68	**(+)**	**(+)**	**(+)**	**(+)**	**(+)**	**(+)**
	T lymphocyte	CD3	**(−)**	ND	ND	**(+)**	**(−)**	ND
		CD4	ND	ND	ND	ND	ND	**(+)**
		CD5	ND	**(+)**	ND	ND	ND	
		CD8	ND	ND	**(−)**	ND	ND	**(+)**
	B lymphocyte	Bcl-2	ND	**(−)**	ND	ND	ND	ND
		Bcl-6	ND	**(−)**	ND	ND	ND	ND
		CD10	ND	**(−)**	ND	ND	ND	ND
		CD20	**(−)**	**(+)**	**(−)**	**(+)**	**(−)**	ND
		CD21	ND	**(−)**	ND	ND	ND	ND
		CD23	ND	**(−)**	ND	ND	ND	ND
	Plasma cells	CD38	ND	ND	ND	ND	ND	**(+)**
		CD138	ND	ND	ND	ND	ND	**(+)**
	Blood vessel	CD34	ND	ND	ND	**(+)**	ND	ND
	Cyclin	CyclinD1	ND	**(−)**	ND	ND	ND	ND
	Cell proliferation index	Ki-65	ND	5%	ND	2%	ND	ND

*ND* not described, (+): positive; (−): negative.

In terms of special staining of nerve biopsy, samples from 2 patients underwent AFB staining, with negative results for both (0.00%, 0/2). Samples from two patients underwent weak acid-fast staining; a positive result was found for 1 patient (50.00%, 1/2), while a negative result was found for the other patient.

In terms of immunohistochemistry (ICH), the following positive results were obtained: (1) nerve-related indicators: myelin basic protein (MBP) (75%, 3/4); neurofilament (NF) (66.67%, 2/3); SRY-related HMG-box 10 (SOX-10) (100.00%, 3/3); Luxol fast blue (LFB) (33.33%, 1/3); and S-100-beta (100.00%, 2/2). (2) Cell-related indicators: macrophage (CD68) (100.00%, 6/6); T cells (CD3, CD4, CD5, and CD8) (50.00%, 3/6); B cells (Bcl-2, Bcl-6, CD10, CD20, CD21, and CD23) (50.00%, 3/6); and plasma cells (CD38 and CD138) (16.67%, 1/6).

Skin pathology analysis was performed for 8 patients (Table 7). The positive results were as follows: AFB, 75.00% (6/8); noninfiltration zone, 25.00% (2/8); lymphocytes, 87.50% (7/8); histiocytes, 62.50% (5/8); foam cells, 37.50% (3/8); and granulomas, 50.00% (4/8). All the skin pathology results supported the diagnosis of Hansen disease.

**Table 7:** Skin biopsy results of confirmed patients with Hansen disease enrolled in this study.

Patients	1	2	3	4	5	6	8	11
Epidermis	No obvious thinning	Thinning	Hyperkeratosis	No obvious thinning	ND	Hyperkeratosis	No obvious thinning	Hyperkeratosis
Noninfiltrating zone	**(+)**	**(+)**	(−)	(−)	ND	(−)	(−)	(−)
Histiocytes	**(+)**	ND	**(+)**	**(+)**	**(+)**	**ND**	**ND**	**(+)**
Lymphocytes	**(+)**	ND	**(+)**	**(+)**	**(+)**	**(+)**	**(+)**	**(+)**
Foam cells	**(+)**	**(+)**	ND	**(+)**	ND	ND	ND	ND
Granuloma	**(+)**	**(+)**	ND	**(+)**	**(+)**	ND	ND	ND
Acid-fast stain	**(+)**	**(+)**	**(+)**	**(+)**	**(+)**	ND	ND	**(+)**

*ND* not described, (+): positive; (−): negative.

### Indexes for differential diagnosis

Other indexes, including indices of metabolism, rheumatism, immunity, nutrition, drugs, toxicity, tumors, infection, physical compression, the blood system, and the nervous system, were screened for the differential diagnosis of Hansen disease from neuropathy (Table 8). The indicators of Rheumatism & Immunity were positive in 37.50% (3/8) patients, involved positive results of Proliferating Cell Nuclear Antigen (PCNA) in case 4, Anti streptolysin O (ASO) in case 10, and Antinuclear antibody (ANA) in case 11, respectively. As an indicator of CNS demyelination, MBP IgG was also increased in Patient 4, and demyelination in the periventricular white matter of the lateral ventricle was detected by magnetic resonance imaging (MRI) in Patient 11. All the other results were negative.

**Table 8:** Indexes for the differential diagnosis of Hansen disease from neuropathy.

Category	Index	Etiology and pathology	Diseases	Reference
Metabolism	Blood glucose, glycosylated hemoglobin, oral glucose tolerance test(OGTT)	Metabolic polyneuropathy	Diabetic neuropathy (GN)	^49^
	Thyroid function(TRAb, TSH, TT3, TT4, FT3, FT4, TU, TG-Ab, TPO-Ab)		Hypothyroidism	^50^
	Blood urea nitrogen(BUN) , creatinine(CREA) , urine analysis		Uremia	^51^
Rheumatism	Anti-streptolysin O(ASO), rheumatoid factor (RF), erythrocyte sedimentation rate (ESR), C-reactive protein (CRP)	Vasculitis peripheral neuropathy	Rheumatoid arthritis (RA)	^52^
Immunity	Antinuclear antibody (ANA) (SmD1, U1-snRNP, P0, Nucleosome, dsDNA, Histone, PCNA, CENP, SS-A/RO 60, SS-A/RO 52, SSB/La, Scl-70, Jo-1, AMA-M2, PM-Scl, Mi-2, Ku		Systemic lupus erythematosus (SLE)	^53^
	Liver function, Autoimmune Hepatitis (AIH) antibodies, [Antinuclear antibody (ANA) anti-mitochondrial antibody (AMA-M2) Antiheart Antibody (AHA) anti-smoothmuscle antibody (SMA) anti-gastric parietal cell antibody (APCA) anti-liver-kidney microsomal antibody (LMK)]		Autoimmune hepatitis (AIH)	^54^
	Autoimmune encephalitis antibody, (AMPAR1, AMPAR2, CASPR2, DPPX, GABABR, IgLON5, LGI1, NMDAR)		Autoimmune encephalitis	^55^
	Anti-neutrophil cytoplasmic antibodies (ANCAs) (P-ANCA, C-ANCA, ANCA-MPO, ANCA-PR3)		ANCA associated systemic vasculitis (AASV)	^56^
	Anticardiolipin antibody (ACA or ACLA) [Anticardiolipin antibody(ACA) , β2-GP1 Ab]		Antiphospholipid syndrome(APS)	^57^
Nutrition	Vitamin B1, Vitamin B6, Vitamin B12, Vitamin E	Nutrient deficiency peripheral neuropathy	Vitamin deficiency	^58^
Drug	Drug use of furans	Drug peripheral neuropathy	High dose of furans	^59^
	Drug use of isoniazid		High dose of isoniazid	
	History of exposure to organophosphorous pesticides		Organophosphorus pesticides	
Toxicity	Arsenic (As), Cadmium (Cd), Chromium (Cr), Copper (Cu), Mercury (Hg), Lead (Pb), and Thallium (TI)	Toxicity	As, Pb, Hg, and/or TI poisoning	^60, 61^
Tumour	Tumor markers (AFP, CEA, CA15-3, CA19-9, CA125, CA72-4, T-PSA, F-PSA, F-PSA/PSA, CYFRA21-1, NSE)	Tumour	Tumor	^62^
	Paraneoplastic neurological syndromes [Antibodies of Amphiphysin, CV2, PNMA2(Ma2/Ta), Ri, Yo, Hu, GAD65,Recoverin, Sox1, Titin, Tr(DNER) , Zic4]		Paraneoplastic neurological syndromes	
Infection	HIV, syphilis, hepatitis B, hepatitis C	Infectious peripheral neuropathy	HIV, syphilis, etc	^63, 64^
	Brucella, Lyme antibody, TORCH [T (Toxoplasma), R (Rubella.Virus), C (Cytomegalovirus), H (Herpes.Virus)]		Brucella, *Borrelia burgdorferi*, etc	^65, 66^
	Bacteria, fungi, cryptococcus, tuberculosis,		Bacteria, fungi, cryptococcus, tuberculosis,	^3, 67^
Physical compression	Cervical, thoracic, lumbar disc herniation	Physical compression causes peripheral nerve involvement	Cervical spondylosis, thoracic spondylosis, lumbar spondylosis	^68^
Blood system	Immunoelectrophoresis (M protein), immunofixation electrophoresis (kappa, LAMDA chain)	Malignant proliferation of monoclonal plasma cells in bone marrow	Myeloma with peripheral neuropathy	^69^
Nervous system	Ganglioside antibody profile (IgG and IgM of Sulfatide, GQ1b, GT1b, GT1a, GD3, GD2, GD1b, GD1a, GM4, GM3, GM2, and GM1)	Immune attack nervous system	Anti-ganglioside antibody-mediated neuropathies, Guillain-Barré syndrome, etc	^70^
	Nodes of Ranvier antibodies (CASPR1, CASPR2, Contactin-1 and Contactin-2, MAG, NF155, NF186, NrCAM, gliomedin)	Immune attack of Nodes of Ranvier	Nodes of Ranvier neuropathy	^71,72,73^
	Central nervous system demyelinating antibodies (IgG of NMO-AQP4, MOG, MBP)	Inflammatory of nervous system	Chronic inflammatory demyelinating polyneuropathy (CIDP)	^74,75^

### Hansen disease symptom monitoring criteria

All patients with Hansen disease were evaluated with the Hansen disease Symptom Monitoring criteria (Supplementary Table S1), and those meeting the criteria of suspected Hansen disease were transferred from the neurology departments to the Hansen disease prevention facility.

### Physical examination by Hansen disease prevention specialists

Skin lesions, nerve lesions, and disability grades were evaluated by Hansen disease prevention specialists (Table 9). Skin lesions occurred in 81.82% (9/11) of the patients. For 2 patients without obvious skin lesions, skin numbness was found in 1 patient, and a deceased prickle sensation was found in the other patient.

**Table 9:** The confirmed diagnosis results of the patients with Hansen disease enrolled in this study.

Patient			1	2	3	4	5	6	7	8	9	10	11
Endemic Regions			Shaanxi	Anhui	Heilongjiang	Hunan	Sichuan	Hubei	Yunnan/ Hebei	Hubei	Jilin	Shaanxi	Sichuan/ Xinjiang,
			Middle	Middle	Low	High	High	High	High/Low	High	Low	Middle	High/Low
Contact History			Absent	Absent	Absent	Absent	Absent	Absent	Present	Absent	Absent	Absent	Absent
Physical examination													
Skin Lesion (n)			≧5	≧5	≧5	≧5	≧5	≧5	1	(2–4)	0	0	≧5
Nerve Lesion (n)			≧2	≧2	≧2	≧2	≧2	≧2	≧2	≧2	≧2	≧2	≧2
Nerve palpation			L/R	L/R	L/R	L/R	L/R	L/R	L/R	L/R	L/R	L/R	L/R
Nerve Tenderness	Supraorbital		(+ / +)	(− / −)	(− / −)	(− / −)	(− / −)	(− / −)	(− / −)	(− / −)	(− / −)	(− / −)	(− / −)
	Greater auricular		(− / −)	(− / −)	(− / −)	(− / −)	(− / −)	(− / −)	(− / −)	(− / −)	(− / −)	(− / −)	(− / −)
	Ulnar		(− / −)	(+ / +)	(− / −)	(+ /-)	(− / −)	(+ / +)	(− / −)	(− / −)	(+ / +)	(+ / +)	(− / −)
	Median		(+ / +)	(− / −)	(− / −)	(− / −)	(− / −)	(− / −)	(− / −)	(− / −)	(+ / +)	(− / −)	(− / −)
	Radial		(− / −)	(+ / +)	(− / −)	(− / −)	(− / −)	(+ / +)	(− / −)	(-/ +)	(− / −)	(+ / +)	(− / −)
	Common peroneal		(− / −)	(+ / +)	(− / −)	(− / −)	(− / −)	(+ / +)	(− / −)	(− / −)	(+ / +)	(+ / +)	(− / −)
	Tibial		(− / −)	(+ / +)	(− / −)	(+ / +)	(− / −)	(+ / +)	(− / −)	(− / −)	(+ / +)	(+ / +)	(− / −)
Nerve Thickness	Supraorbital		(+ / +)	(− / −)	(− / −)	(− / −)	(− / −)	(− / −)	(− / −)	(− / −)	(− / −)	(− / −)	(− / −)
	Greater auricular		(+ / +)	(− / −)	(− / −)	(− / −)	(− / −)	(− / −)	(− / −)	(− / −)	(− / −)	(− / −)	(− / −)
	Ulnar		(+ / +)	(-/ +)	(− / −)	(+ / +)	(+ / +)	(− / −)	(− / −)	(− / −)	(− / −)	(− / −)	(− / −)
	Median		(− / −)	(− / −)	(− / −)	(− / −)	(− / −)	(− / −)	(− / −)	(− / −)	(− / −)	(− / −)	(− / −)
	Radial		(− / −)	(− / −)	(− / −)	(− / −)	(− / −)	(− / −)	(− / −)	(-/ +)	(− / −)	(− / −)	(− / −)
	Common peroneal		(− / −)	(− / −)	(− / −)	(− / −)	(− / −)	(− / −)	(− / −)	(− / −)	(− / −)	(− / −)	(− / −)
	Tibial		(+ / +)	(− / −)	(+ / +)	(+ / +)	(− / −)	(− / −)	(− / −)	(− / −)	(− / −)	(− / −)	(− / −)
Grade of disability			G2D	G1D	G2D	G2D	G2D	G2D	G2D	G2D	G1D	G2D	G2D
G1D	Insensitivity		(+)	(+)	(+)	(+)	(+)	(+)	(+)	(+)	(+)	(+)	(+)
G2D	Lagophthalmos		(−)	(−)	(−)	(−)	(+)	(−)	(−)	(−)	(−)	(−)	(−)
	Claw hand		(+)	(−)	(−)	(−)	(+)	(+)	(+)	(−)	(−)	(−)	(−)
	Complex plantar ulcer		(−)	(−)	(−)	(−)	(−)	(+)	(−)	(−)	(−)	(−)	(−)
	Amputation		(−)	(−)	(−)	(−)	(−)	(+)	(−)	(−)	(−)	(−)	(−)
	Foot drop		(−)	(−)	(−)	(+)	(−)	(+)	(−)	(−)	(−)	(+)	(+)
	Muscle atrophy		(−)	(−)	(+)	(−)	(−)	(+)	(+)	(−)	(−)	(−)	(−)
	Wrist drop		(−)	(−)	(−)	(−)	(−)	(−)	(−)	(+)	(−)	(−)	(−)
	Equinus		(−)	(−)	(−)	(+)	(−)	(−)	(−)	(−)	(−)	(−)	(−)
Laboratory tests													
AFB	Skin		(+)	(+)	(+)	(+)	(+)	(−)	(−)	(−)	(−)	(−)	(+)
	Nerve		ND	ND	ND	ND	(+)	ND	ND	ND	ND	ND	(−)
Skin pathology			Confirmed	Confirmed	Confirmed	Confirmed	Confirmed	Confirmed	ND	Confirmed	ND	ND	Confirmed
Serology	NDO-LID Rapid Test		(+)	(+)	(−)	(+)	(−)	(−)	(−)	(−)	(−)	(−)	(-)
	ELISOPT(IFN-r)		ND	ND	ND	ND	ND	ND	ND	ND	(+)	ND	ND
Molecular technology	Nasal Swabs	RLEP	(+)	(+)	(+)	(+)	(−)	(−)	(+)	(−)	(−)	(−)	(−)
		RpoB	( ±)	(+)	(−)	(+)	(−)	(−)	(+)	(−)	(+)	(−)	(−)
		FolP1	(+)	(+)	(−)	(+)	(+)	(−)	(−)	(+)	(+)	(−)	(−)
		GyrA	(+)	(+)	(+)	(+)	(+)	(−)	(+)	(+)	(+)	(+)	(−)
	Skin slit smear	RLEP	(+)	(+)	(+)	(+)	(−)	(+)	(−)	(−)	(+)	(−)	(−)
		RpoB	(+)	(+)	(+)	(−)	(−)	(−)	(−)	(−)	(+)	(−)	(−)
		FolP1	(+)	(+)	(−)	(+)	(+)	(−)	(+)	(−)	(+)	(−)	(+)
		GyrA	(+)	(+)	(+)	(+)	(+)	(−)	(−)	(−)	(+)	(+)	(−)
	Blood	RLEP	(+)	(−)	(−)	(−)	(+)	(−)	(−)	(−)	(−)	(−)	(−)
		RpoB	(+)	(+)	(−)	(−)	(−)	(−)	(−)	(−)	(−)	(−)	(−)
		FolP1	(+)	(−)	(−)	(+)	(+)	(−)	(−)	(−)	(+)	(+)	(−)
		GyrA	(+)	(+)	(−)	(+)	(+)	(−)	(+)	(−)	(−)	(−)	(−)
	Skin biopsy	RLEP	(+)	(+)	(−)	(+)	(+)	(+)	ND	(−)	ND	ND	(−)
		RpoB	(+)	(+)	(−)	(−)	(−)	(+)	ND	(−)	ND	ND	(+)
		FolP1	(+)	(−)	(−)	(+)	(+)	(−)	ND	(−)	ND	ND	(+)
		GyrA	(+)	(+)	(−)	(+)	(−)	(−)	ND	(+)	ND	ND	(+)
	Nerve biopsy	RLEP	ND	ND	ND	ND	ND	ND	ND	ND	ND	(+)	NGS (+)
		RpoB	ND	ND	ND	ND	ND	ND	ND	ND	ND	(−)	
		FolP1	ND	ND	ND	ND	ND	ND	ND	ND	ND	(+)	
		GyrA	ND	ND	ND	ND	ND	ND	ND	ND	ND	(+)	
Neurological examinantion													
Nerve ultra-sonography	Ulnar		ND	ND	ND	ND	**(+ / +)**	**(−/ +)**	**(ND/ +)**	**(−/ +)**	**(−/ +)**	ND	**(+ / +)**
	Median		ND	ND	ND	ND	**(+ / +)**	**(−/ +)**	**(+ / +)**	**(−/ +)**	ND	ND	**(+ / +)**
	Radial		ND	ND	ND	ND	ND	ND	ND	**(−/ +)**	ND	ND	ND
	Common peroneal		**(+ / +)**	ND	**(+ /−)**	ND	ND	ND	**(+ /−)**	**(−/−)**	**(−/−)**	**(−/ +)**	ND
	Tibial		**(+ / +)**	ND	ND	ND	ND	ND	ND	**(−/−)**	ND	**(−/ +)**	ND
Electro-diagnostic study	NCV		Sensory	ND	Sensory	Sensory	Sensory	Sensory	Sensory	Sensory	Sensory	Sensory	Sensory
			Motor		Motor	Motor	Motor	Motor	Motor	Motor	Motor	Motor	Motor
	F wave		No response	ND	Prolonged latency	Normal	Normal	Normal	Normal	Normal	Normal	Normal	Prolonged latency
													Decreased freuency
	H reflection		Normal	ND	Normal	Normal	ND	ND	Normal	Normal	Normal	Normal	ND
	SSR		No Response	ND	ND	ND	ND	ND	No Response	ND	ND	ND	ND
	EMG		Neurogenic damage	ND	Neurogenic damage	Neurogenic damage	Neurogenic damage	Neurogenic damage	Neurogenic damage	Neurogenic damage	Neurogenic damage	Neurogenic damage	Neurogenic damage
Peripheral nerve study	Nerve pathology		ND	ND	ND	ND	Demyelination	Demyelination	Demyelination	ND	ND	Demyelination	Demyelination
							Axonal damage	Axonal damage	Axonal damage			Axonal damage	Axonal damage
Indexes for differential diagnosis													
Rheumatism & Immunity			(−)	(−)	(−)	PCNA(+)	(−)	(−)	(−)	(−)	(−)	ASO(+)	ANA(+)
CNS	NMO-AQP4 IgG		ND	ND	ND	Normal	ND	ND	ND	ND	ND	ND	(−)
	MOG IgG		ND	ND	ND	Normal	ND	ND	ND	ND	ND	ND	(−)
	MBP IgG		ND	ND	ND	0.69 nmol/L↑	ND	ND	ND	ND	ND	ND	(−)
	MRI		ND	ND	ND	ND	ND	ND	ND	ND	ND	ND	Demyelination in the periventricular white matter of the lateral ventricle
Confirmed diagnosis													
Classification			New	New	New	New	New	New	New	New	New	New	Relapsed
Ridley-Jopling			LL	BL	BL	BB	BB	BT	TT	TT	PNL	PNL	/
WHO			MB	MB	MB	MB	MB	PB	PB	PB	PB	PB	/

*G1D* grade 1 disability, *G2D* grade 2 disability, *AFB* acid fast bacilli, *NCV* nerve conduction velocity, *NGS* next-generation sequencing, *LL* lepromatous leprosy, *BL* boderline leprosy, *BB* boderline lerosy, *BT* boderline tuberculoid leprosy, *TT* tubuloid leprosy, *PNL* pure neuritic leprosy. *CNS* Central nervous system, *MRI* magnetic resonance imaging, *ND* not done, *L/R* left/right.

Nerve lesions occurred in all patients (100.00%, 11/11) and presented as more than 2 nerve lesions (Table 9). All of the patients (100.00%, 11/11) were evaluated for nerve thickening and nerve tenderness by nerve palpation, and the percentage of patients with nerve tenderness was 33.12% (51/154), which was greater than that with nerve thickening (12.34%, 19/154). (P < 0.05) (Table 5). The nerves most affected by tenderness were the tibial (54.54%, 10/22), ulnar (40.90%, 9/22), common peroneal (31.81%, 7/22), radial (22.72%, 5/22), medial (22.72%, 5/22), and supraorbital (9.09%, 2/22) nerves, but no tenderness of the greater auricular nerve was detected (0.00%, 0/22). The nerves most affected by thickening were the tibial (31.81%, 7/22), ulnar (31.81%, 7/22), supraorbital (9.09%, 2/22), greater auricular (9.09%, 2/22), and radial (4.54%, 1/22) nerves; however, no median (0.00%, 0/22) or common peroneal (0.00%, 0/22) nerve thickening was detected.

Twenty-one nerves were tested by both nerve ultrasonography and nerve palpation: [33.33% (7/21) *vs* 9.52% (2/21), P = 0.1595], [52.38% (11/21) *vs* 4.76% (1/21), P = 0.0171*] and [14.29% (3/21) *vs* 9.52% (2/21), P > 0.9999] of the nerves showed bilateral, unilateral, and a lack of nerve thickening according to nerve ultrasonography and nerve palpation, respectively. The positive finding of unilateral nerve thickness by nerve ultrasound were significantly higher than those by nerve palpation.

Disability occurred in all of the patients (100.00%, 11/11) (Table 9). Grade 2 disability (G2D) occurred in 81.81% (9/11) of patients, while grade 1 disability (G1D) occurred in 18.18% (2/11) of patients. Insensitivity occurred in all of the patients (100.00%, 11/11), claw-hand motion occurred in 36.36% (4/11) of the patients, foot droppage occurred in 36.36% (4/11) of the patients, and muscle atrophy occurred in 27.27% (3/11) of the patients. In addition, 9.09% (1/11) of the patients presented with lagophthalmos, 9.09% (1/11) presented with equinus, and 9.09% (1/11) presented with complex plantar ulcers, amputation, and wrist drop.

### Auxiliary laboratory Hansen disease tests

For auxiliary laboratory Hansen disease tests, AFB staining and molecular tests were performed in all 11 patients: AFB staining was positive in 54.54% (6/11) patients, while molecular test [Nested PCR for Repetitive element (RLEP); dapsone resistance—associated target (folP1); rifampicin resistance-associated target (rpoB); quinolone resistance—associated target (gyrA), confirmed as *M.leprae* by sequencing and NGS] results were positive in 100.00% (11/11) patients. Skin pathology analysis, the “gold standard” for diagnosis of Hansen disease, was performed for 8 of the 11 (63.63%) patients, and the diagnosis of Hansen disease was confirmed for all the patients (8/8, 100.00%). Among the serological tests, the NDO-LID rapid test was performed for 11 patients, and results were positive for 36.36% (3/11) of the patients. In addition, ELISPOT was performed in only 1 patient (9.09%, 1/11), and a positive result was achieved (1/1, 100.00%) (Table 9).

### Confirmed diagnosis of Hansen disease

Hansen disease was confirmed in eleven patients, including 10 patients with newly detected and 1 patient with relapse, according to the diagnostic criteria for Hansen disease in WHO^10^. The details are shown in Table 9.

### Deteriorated disability due to delayed diagnosis

In this study, delayed diagnosis and G2D occurred in 90.90% (10/11) and 81.81% (9/11) of the patients, respectively. Multiple medical records of Hansen disease patients revealed worsening disability (100.00%, 3/3) (Table 10), decreased muscle strength (100.00%, 3/3) (Table 11), worsening nerve conduction (66.66%, 3/3) (Table 12) and worsening nerve ultrasonography findings (100.00%, 1/1) (Table 13) due to delayed diagnosis.

**Table 10:** Worsening disability in the multiple medical records of patients with Hansen disease.

Patient 6	GD	Disability and deformity	2004y	2005y	2008y	2008y	2010y	2012y	2021y
	G1D	Insensitivity	Right hand	Right hand	Right hand	Right hand	Right hand	Right hand	Right hand
				Right Foot	Right Foot	Right Foot	Right Foot	Right Foot	Right Foot
					Left hand	Left hand	Left hand	Left hand	Left hand
						Left foot	Left foot	Left foot	Left foot
	G2D	Complex plantar ulcer					Right Foot		
		Amputation						Right Foot	
		Foot drop							Right Foot
	GD	Grade of disability (GD)				G1D	G2D		
Patient 7	GD	Disability and deformity				2017y	April, 2020	May,2020	November,2020
	G1D	Insensitivity				Right hand	Right hand	Right hand	Right hand
								Lower limb	Lower limb
	G2D	Claw hand					Right hand	Right hand	Right hand
	GD	Grade of disability (GD)				G1D	G2D		
Patient 11	GD	Disability and deformity					2018y	2019y	2023y
	G1D	Insensitivity					Both lower limbs	Both lower limbs	Both lower limbs
								Left hand	Left hand
									Right hand
	G2D	Foot drop						Left Foot	Left Foot
	GD	Grade of disability (GD)					G1D	G2D

**Table 11:** Muscle strength decreased according to the medical records of the patients with Hansen disease.

Patient		Patient 6								Patient 7						Patient 11					
Period		16 y ago		10 y ago		8 y ago		0 y		3 y ago		0.5 y ago		0 y		5 y ago		0.5 y ago		0 y	
Muscle strength		L	R	L	R	L	R	L	R	L	R	L	R	L	R	L	R	L	R	L	R
Upper limbs	Shoulder abduction	V	V	**V–**	**V–**	V	**V–**	V	V	V	V	V	V	V	V	V	V	V	V	V	V
	Elbow flexion	V	V					V	V	V	V	V	V	V	V	V	V	V	V	V	V
	Elbow extension	V	V					V	V	V	V	V	V	V	V	V	V	V	V	V	V
	Wrist flexion	V	V	**V–**	**V–**	**III**	**III**	V	V	V	V	V	V	V	V	V	V	V	V	V	V
	Wrist extension	V	V					V	V	V	V	V	V	V	V	V	V	V	V	V	V
	Finger flexion	V	V					V	**IV**	V	V	V	V	V	**III**	V	V	V	V	V	V
	Finger extension	V	V	**V–**	**V–**			V	V	V	V	V	V	V	III	V	V	V	V	V	V
	Fingers apart	V	V					**III**	**I**	V	V	V	**IV**	V	**IV**	V	V	V	V	V	V
	Fingers together	V	V					**III**	**I**	V	V	**IV**	**IV**	**IV**	**IV**	V	V	V	V	V	V
	Froment's Sign	(−)	(−)	(−)	(−)	(−)	(−)	**(+)**	**(+)**	(−)	(−)	**(+)**	**(+)**	**(+)**	**(+)**	(−)	(−)	(−)	(−)	(−)	(−)
Lower limbs	Hip flexion	V	V	**V–**	**V–**	V	V	V	V	V	V	V	V	V	V	V	V	**IV**	**IV**	**V–**	**V–**
	Hip extension	V	V					V	V	V	V	V	V	V	V	V	V				
	Bend flexion	V	V					V	V	V	V	V	V	V	V	V	V				
	Bend extension	V	V					V	V	V	V	V	V	V	V	V	V				
	Dorsalis Pedis flexion	V	V	**V–**	**V–**	V	V	V	**I**	V	V	**IV**	V	**IV**	V	V	V	**IV**	**IV**	**V–**	**V–**
	Plantar flexion	V	V					V	**I**	V	V	**IV**	V	**IV**	V	V	V				
	Toe flexion	V	V					V	V	V	V	**IV**	V	**IV**	V	V	V				
	Toe extension	V	V					V	V	V	V	**IV**	V	**IV**	V	V	V				
	Steppage gait	(−)		(−)		(−)		**(+)**		(−)		(−)		(−)		(−)		(−)		(+)	

Significant values are in bold.

(+): positive; (−): negative.

**Table 12:** Deteriorated nerve conduction in the Multiple medical records of the patients with Hansen disease.

Nerve conduction velocity		Motor			Sensory			Motor			Sensory		
		Latency (m/s)	Amplitude (mV)	Conduction velocity (m/s)	Latency (m/s)	Amplitude (mV)	Conduction velocity (m/s)	Latency (m/s)	Amplitude (mV)	Conduction velocity (m/s)	Latency (m/s)	Amplitude (mV)	Conduction velocity (m/s)
Patient 6		Early stage (2014)						Advanced stage (2022)					
Ulnar	Left	ND	ND	ND	ND	ND	ND	Normal	Normal	Normal	Normal	Normal	Normal
	Right	ND	ND	ND	ND	ND	ND	No Response			No Response		
Median	Left	ND	ND	ND	ND	ND	ND	Normal	Normal	Normal	Normal	Normal	Normal
	Right	Normal	5.6↓	47↓	Normal	14.3↓	36↓	Normal	0.07↓ 99%	Normal	No Response		
Radial	Left	ND	ND	ND	ND	ND	ND	ND	ND	ND	ND	ND	ND
	Right	Normal	Normal	Normal	Normal	Normal	Normal	Normal	Normal	Normal	No Response		
Common peroneal	Left	ND	ND	ND	ND	ND	ND	No Response			No Response		
	Right	Normal	Normal	Normal	Normal	Normal	Normal	No Response			No Response		
Tibial	Left	ND	ND	ND	ND	ND	ND	Normal	Normal	Normal	No Response		
	Right	Normal	6.1↓	35↓	ND	ND	ND	ND	ND	ND	ND	ND	ND
Patient 7		Early stage (2017)						Advanced stage (2020)					
Ulnar	Left	ND	ND	ND	ND	ND	ND	Normal	Normal	Normal	Normal	Normal	Normal
	Right	No Response			No Response			Normal	1.815↓ 90%	Normal	No Response		
Median	Left	ND	ND	ND	ND	ND	ND	Normal	Normal	Normal	Normal	Normal	Normal
	Right	–	–	–	No Response			Normal	Normal	Normal	No Response		
Radial	Left	ND	ND	ND	ND	ND	ND	ND	ND	ND	ND	ND	ND
	Right	ND	ND	ND	ND	ND	ND	ND	ND	ND	ND	ND	ND
Common peroneal	Left	No Response			No Response			6.1↑133%	0.047↓ 99%	27↓ 57%	No Response		
	Right	Normal	Normal	Normal	No Response			11.5↑ 339%	1.074↓ 80%	Normal	No Response		
Tibial	Left	Normal	Normal	Normal	No Response			6.2↑59%	1.491↓ 92%	Normal	No Response		
	Right	Normal	Normal	Normal	No Response			5.2↑ 33%	3.356↓ 82%	Normal	No Response		
Patient 11		Early stage (2018)						Advanced stage (2022)					
Ulnar	Left	Normal	Normal	Normal	No Response			Normal	Normal	30.3 m/s↓ 47%	No Response		
	Right	Normal	Normal	Normal	No Response			Normal	Normal	Normal	Normal	2.4↓87%	Normal
Median	Left	Normal	Normal	Normal	No Response			Normal	Normal	Normal	Normal	3.2↓84%	36.6↓76%
	Right	Normal	Normal	Normal	No Response			Normal	Normal	Normal	Normal	5.0↓88%	39.9↓27%
Radial	Left	ND	ND	ND	ND	ND	ND	ND	ND	ND	ND	ND	ND
	Right	ND	ND	ND	ND	ND	ND	ND	ND	ND	ND	ND	ND
Common peroneal	Left	No Response			No Response			No Response			No Response		
	Right	Normal	1.7↓	Normal	No Response			4.86↑54%	1.30↓77%	36.6↓76%	No Response		
Tibial	Left	No Response			No Response			6.16↑58%	0.24↓98%	Normal	No Response		
	Right	Normal	Normal	Normal	No Response			Normal	Normal	Normal	No Response		

*ND* not described. Up arrow (↑): increased; down arrow (↓): decreased.

**Table 13:** Deteriorated nerve ultrasound in the multiple medical records of the patients with Hansen disease.

Nerve ultrasonography Patient 7		2020.04.24	2020.05.14	2020.11.27
CSA (mm^2^)	Ulnar	ND	ND	ND
		φ4.0 (Elbow, Right, ↑)	ND	ND
	Median	φ2.5 (Wrist, Left, Ref) φ3.8 (Wrist, Right↑)	5.1 × 1.4 × 2.9 (Proximal, Wrist, Ref) 7.3 × 3.0 × 54.8 (Distal, Wrist,↑)	10–16 (Left, Ref) 8–19 (Right, ↑)
			5.4 × 1.9 × 3.6 (Proximal, Elbow, Ref) 4.4 × 3.6 × 80.3 (Distal, Elbow,↑)	
	Sciatic nerve	ND	ND	74–136 (Ref)
		ND	ND	80–139
	Common peroneal	ND	ND	19–35 (↑)
		ND	ND	16–26 (Ref)
Asymmetrical and segmental thickening		**(+)**	**(+)**	**(+)**
Nerve swelling		ND	ND	**(+)**
Echo intensity	Nerve bundles	ND	ND	↓
	Epineurium	ND	ND	ND
Continuity		ND	ND	Good
CDFI		ND	ND	No

*CSA* cross-sectional area, *CDFI* color blood flow signal, *ND* not described.

Up arrow (↑): increased; down arrow (↓): decreased. φ diameter.

## Discussion

Hansen disease is a chronic infection caused by *Mycobacterium leprae* that is associated with peripheral neuropathy. Early Hansen disease detection and treatment with multidrug therapy are the most important steps in preventing deformity and disability^11^.

The gold standard for Hansen disease diagnosis is dermatological and neurological clinical examination, bacilloscopy/AFB staining of the SSS and skin biopsy sample analysis^12,13^. To confirm the diagnosis, laboratory tests, AFB staining, skin and nerve biopsy, and serological and molecular tests were performed for all of the patients. In this study, the diagnoses of 11 patients with Hansen disease were confirmed: as 1 LL, 2 BL, 2 BB, 1 BT, 2 TT, 2 PNL, and 1 relapsed. The diagnoses of six (1 LL, 2 BL, 2 BB and 1 relapsed) patients were confirmed by AFB staining, and the diagnoses of 8 (1 LL, 2 BL, 2 BB, 1 BT, 1 TT, and 1 relapsed) patients were confirmed by skin biopsy. AFB staining results were negative in 1 TT and 2 PNL patients, and skin biopsy was not suitable for some TT or PNL patients, which implies the limited diagnostic value of traditional laboratory tests for Hansen disease. Notably, the nerve biopsy sample of 1 (BB) patient was positive for AFB staining, which provided morphological evidence of *Mycobacterium leprae* infection in the in situ nerve tissue.

Since the 1980s, components isolated from *M. leprae* have been identified^14,15^; an increasing number of experimental trials have used these components to detect antibodies in Hansen disease patients. These detection methods are mainly used for diagnosing MB of Hansen disease, monitoring treatment response, and predicting leprosy reactions and as active search strategies to identify new cases in high-risk populations^16–19^.

In the past three decades, definitive identification of *M. leprae* has been possible through the development of methods for the extraction, amplification, and identification of *M. leprae* DNA in clinical specimens via PCR. PCR has been ascertained to be especially valuable in diagnosing difficult cases, such as those with PNL, PB, and atypical clinical presentations and histopathological features compatible with Hansen disease^20^. These methods were also used in this study, and the positive results supported the diagnosis of Hansen disease, especially for TT and PNL patients.

In this study, serological (NDO-LID rapid test and ELISPOT) and molecular [multitarget genes, PCR, Sanger sequencing, and next-generation sequencing (NGS) in multiple specimens] methods were used for the detection of *M. leprae*. These methods successfully detected *M. leprae* and supported the diagnosis of Hansen disease, including in patients with tuberculoid forms and PNL. NDO-LID rapid test results were positive in 3 lepromatous form (1 LL, 1 BL, and 1 BB) patients, which implied that the method has limited value for the auxiliary diagnosis of tuberculoid form and/or PNL. However, the ELISPOT showed greater diagnostic value for tuberculoid form of Hansen disease, as it was performed in 1 patient with suspected Hansen disease without skin lesions, and the positive result supported the diagnosis of PNL. Notably, positive molecular signals were detected via nerve biopsy in 2 patients with Hansen disease (1 patient with PNL as determined by Sanger sequencing and 1 patient with a relapse as determined by NGS), which provided etiological evidence of *Mycobacterium leprae* infection of nerve tissue.

To confirm the diagnosis of Hansen disease, an endemic region, contact history, skin and nerve examination, AFB staining, and skin pathology analysis were performed for all patients. Six, three and two patients from high-, middle-, and low-endemic regions, respectively, were diagnosed with Hansen disease in China. One patient had a contact history of Hansen disease, and the other patient, a clinically cured patient, had neurologic symptoms and signs of Hansen disease onset after multidrug therapy (MDT) was completed. The remaining patients had no contact history of Hansen disease. Notably, (1) Two patients with Hansen disease, as the floating population, moved from a high-endemic region to a low-endemic region of Hansen disease in China (from Yunnan to Hebei, and from Sichuan to Xinjiang, respectively). This was in consistent with another study in China^21^, which implied Hansen disease patients in the floating population overflowed from high-endemic areas to low-endemic areas. (2) All 11 patients with confirmed Hansen disease were from provinces other than Beijing, the capital of China, and most of them experienced multiple hospital visits, delayed diagnosis and permanent disability and deformity. This implies that Hansen disease patients with difficult cases were transferred to areas with medical advantages according to their medical needs.

The clinical characteristics of patients with Hansen disease in neurology departments have mostly been described in case reports, with diagnoses based on a single technology, and overall knowledge of the neurological features of Hansen disease is still limited. To elucidate the neurological characteristics of Hansen disease, nerve ultrasonography, EDX, and nerve biopsy findings were thoroughly evaluated.

Nerve ultrasonography has been performed to assess peripheral nerves in patients with Hansen disease in multiple studies. A significant increase in the CSA of the ulnar, median, radial, common peroneal, and tibial nerves of Hansen disease patients has been mostly reported^22–29^. The sonographic findings of Hansen disease patients are characterized by an increased CSA and a pattern of asymmetry and focality of this thickening, with high sensitivity and specificity for early diagnosis^24^. In our previous study, we also observed a combination of an enlarged CSA of nerves in the upper limbs and atrophy of lower limb nerves in clinically cured Hansen disease patients^30^. In addition to an abnormal CSA, a loss of fascicles, hypoechogenicity and increased neural vascularity have also been reported in patients with Hansen disease^22^. In this study, asymmetrical and segmental thickening, swelling, and abnormal signs of echo intensity and blood flow were detected via nerve ultrasonography. The US results in our study are consistent with those of previous reports.

The risk factors for nerve enlargement in patients with Hansen disease were MB leprosy^25^, leprosy reaction^26^, neuritis^27^, and impairment of function^28^. Nerve ultrasonography for Hansen disease can be applied in the early diagnosis of Hansen disease in household contacts (HHCs)^29^ and for monitoring the therapeutic effects of MDT^27^. Compared with healthy volunteers, individuals with at least two thickened nerves assessed in the active search campaign had a 23.1 greater chance of having Hansen disease than healthy individuals^24^. Nerve ultrasonography showed greater sensitivity than clinical examination for detecting peripheral nerve thickening in Hansen disease patients, which may therefore improve the sensitivity of the diagnostic criterion of peripheral nerve enlargement in the diagnosis and classification of Hansen disease^28^. In this study, we also demonstrated that, when compared with the results of nerve ultrasonography, nerve palpation lacks precision in detecting nerve thickness. Due to the very limited accuracy of nerve palpation, the systematic and comprehensive development of nerve ultrasonography for both Hansen disease patients and close contacts is needed.

Abnormal nerve conduction in patients with Hansen disease was characterized by reduced conduction velocities in addition to changes in prolonged distal latency and decreased amplitude in the affected nerves^31–33^. Sensory latency and amplitude changes were more severe than motor latency and amplitude changes in patients presenting with muscle palsies^32,33^. Patients with the TT type of Hansen disease were the most affected^33,34^. Electrophysiological testing showed both axonal and demyelinating nerve involvement^35,36^.

The number of nerve abnormalities detected by electrophysiological testing is significantly greater than that detected clinically^36^. In Hansen disease patients, motor weakness, sonographic thickening, and motor conduction abnormalities are positively correlated^37^. An approach combining nerve ultrasonography and electrophysiological testing can improve Hansen disease diagnosis^38^.

Nerve histology is studied less often than cutaneous histology. Depending upon the host immune response, a spectrum of pathological changes in the skin are reflected in nerves. At the tuberculoid end of the spectrum, epithelioid granulomas with little or no AFB staining are observed, while at the lepromatous end, abundant AFB staining of Schwann cells, macrophages and plasma cell infiltrates are observed^39^. Other nerve changes include mild perineural edema, partial involvement of fascicles, a loss of fiber density and areas of demyelination^40^. Demyelination can occur primarily at the site of acute neuritis or secondary to chronic axonopathy. In severe cases, there is destruction of the nerve parenchyma with caseous necrosis with epithelioid infiltrates and segmental necrotizing granulomatous neuritis^41^. During spontaneous or posttreatment regression of nerve lesions, residual signs of chronic inflammation with lymphocytic infiltration and evidence of regeneration and fibrosis are observed^42^. The results of nerve biopsy in this study were consistent with those of previous studies.

Along with peripheral nerves, the central nervous system (CNS), spinal root ganglion and brachial plexus are involved in Hansen disease^43,44^. MBP, an indispensable protein of myelinated axons, is abundant in CNS myelin and has long been studied as a factor in the pathogenesis of neurodegenerative diseases, such as multiple sclerosis (MS)^45^ and reported in Hansen disease^46^. In this study, demyelination in the periventricular white matter of the lateral ventricle was detected by MRI in one patient, and MBP IgG was elevated in another patient. This finding provides evidence of demyelination in the CNS and peripheral nervous system in patients with Hansen disease.

## Conclusion

Hansen disease patients may visit the neurology department due to neurological manifestations. Patients with peripheral neuropathy with or without skin lesions, after excluding other causes, were considered suspected to have Hansen disease.

The ultrasonographic features of Hansen disease included asymmetrical and segmental patterns of increased CSA. The electrophysiological features included no response, reduced conduction velocities, prolonged latency and decreased amplitude in the affected nerves. Nerve pathological features included AFB staining; axon and myelin sheath destruction; Schwann cell hyperplasia or an absence of macrophage, plasma cell, and/or lymphocyte infiltration; fibrosis; and edema.

The combination of ultrasonographic and electrophysiological testing with serological and molecular testing may improve the diagnosis of Hansen disease. Nerve biopsy can be chosen for difficult cases of PNL when the diagnosis is unclear and to provide in situ evidence for the pathogen in nerve tissue.

## Methods

### Ethics statement

This study was approved by the Medical Ethics Committee of Beijing Friendship Hospital, Capital Medical University, Beijing, P. R. China. Written informed consent was obtained from all adult participants. All the procedures that involved human participant selection were performed in accordance with the ethical standards of the institutional and/or national research committee and Declaration of Helsinki, 1964, and its later amendments or comparable ethical standards.

### Hansen disease patients

This retrospective analysis was carried out at multiple centers. All the patients with suspected Hansen disease were transferred by neurologists from the neurology departments of general hospitals to the Hansen disease prevention facility. Patients with suspected Hansen disease for whom Hansen disease diagnosis was confirmed between January 1, 2018, and September 1, 2020, were included in this study. The diagnosis of Hansen disease was based on the Leprosy Diagnosis Criteria of WHO^10^.

### Data collection

The medical records of all patients were retrospectively reviewed as follows:

1. The patients’ clinical data, including sex, age, ethnic group, domicile, place of residence, main complaint, disease duration, clinical symptoms, time of appearance of specific symptoms, Hansen disease contact history, physical examination (skin, nerve lesions, and grade of disability), and ancillary test results (AFB staining and skin pathology analysis), were collected from the Leprosy Management Information System in China (LEPMIS).
2. The patients’ neurologic examination results were retrieved from the medical records of the neurology departments of the transfer hospitals. Neurologic examinations were performed by neurologists. Skin lesions were assessed, as were the muscle strength, movement, and sensory impairment of limbs.
3. The features of the ulnar, median, radial, brachial plexus, common peroneal, tibial, and sciatic peripheral nerves were evaluated by nerve ultrasonography. The CSA, swelling, echo intensity of nerve bundles and the epineurium, and the continuity and CDFI of the bilateral nerves were measured via ultrasonography^47,48^.
4. The electrophysiologic features of the peripheral nerves, specifically the ulnar, median, radial, common peroneal, and tibial tract nerves, were analyzed. Motor and sensory NCV, F-wave, H reflex, SSR and EMG were conducted based on standard procedures.
5. The pathological features of the nerves were detected via HE staining, special staining, and ICH in the nerve biopsy.
6. The indexes for the differential diagnosis of Hansen disease from neuropathy were tested, and the results were collected^49–75^.

### Laboratory tests

Clinical specimens, including skin slit smear (SSS), nasal swab, blood, and biopsy specimens, were collected from patients with suspected Hansen disease. AFB staining and histological analyses of biopsy specimens were performed. Procedures for serological and molecular tests, which mainly included the NDO-LID rapid test, Nested-PCR, and the ELISPOT, were described in previous studies^76–80^. The sanger sequencing and next generation sequencing (NGS) were performed by Sangon Biotech (Shanghai) Co., Ltd. and Guangzhou Oumengweiyi Medical Laboratory Co., Ltd., respectively.

### Statistical analysis

Statistical analysis was performed using GraphPad Prism 8.0 software. The demographic data are reported using descriptive statistics, including percentages, means, and standard deviations. The clinical features of male and female patients were compared using the chi-square test. P < 0.05 indicated statistical significance.

### Ethics approval and consent to participate

This study involving human participants was reviewed and approved by the Medical Ethics Committee of Beijing Friendship Hospital, Capital Medical University, Beijing, P.R. Chin. Written informed consent to participate in this study was provided by the participants.

### Supplementary Information

Supplementary Table S1.

## Data Availability

The datasets generated and/or analyzed during the current study are available from the corresponding author upon reasonable request.
